# Neonicotinoid Imidacloprid Affects the Social Behavior of Adult Zebrafish by Damaging Telencephalon Neurons through Oxidation Stress, Inflammation, and Apoptosis

**DOI:** 10.3390/life13061418

**Published:** 2023-06-20

**Authors:** Kou-Toung Chung, Li-Wen Chen, Hung-Wei Tseng, Chung-Hsin Wu

**Affiliations:** 1 Department of Chemical Engineering, Army Academy of ROC, Taoyuan City 320, Taiwan; chungkoutoung@gmail.com; 2 Department of Science Education and Application, National Taichung University of Education, Taichung City 403, Taiwan; liwenchen@mail.ntcu.edu.tw (L.-W.C.); ives0208@hotmail.com (H.-W.T.); 3 School of Life Science, National Taiwan Normal University, Taipei 106, Taiwan

**Keywords:** imidacloprid, oxidative stress, inflammation, apoptosis, telencephalon, zebrafish

## Abstract

The neonicotinoid imidacloprid is a widely used insecticide worldwide. We assessed the effects of acute and chronic imidacloprid exposure on the social behavior of adult zebrafish. We assembled simple apparatus to detect 2D locomotion: a single camera capture system and two specially designed water tanks. We then used the tracking and heat maps of the behavior trajectories of zebrafish subjected to sham and imidacloprid exposure and compared their social behavior. Furthermore, histomorphology and immunohistochemistry of their brain tissue sections were performed to clarify possible neurotoxicity due to imidacloprid exposure in our adult zebrafish. Our results showed that imidacloprid exposure significantly reduced the zebrafish’s swimming speed, distance traveled, acceleration, and deceleration. The longer the imidacloprid exposure, the more severe the locomotor behavior disability. Furthermore, imidacloprid exposure significantly reduced heterosexual attractive behavior between the different sexes, as well as defensive alert behavior among males. Our histomorphology and immunohistochemistry evidence showed imidacloprid exposure may lead to neuronal oxidative stress, inflammation, apoptosis, and damage in the telencephalon of adult zebrafish. Thus, we suggested that neonicotinoid imidacloprid exposure can damage the telencephalon neurons of adult zebrafish through oxidative stress, inflammation, and apoptosis and then affect the social behavior of adult zebrafish.

## 1. Introduction

The neonicotinoid imidacloprid has been widely used as a pesticide because it negatively affects the nervous system of insects. It has an adequate insecticidal effect at a small dose. Consequently, imidacloprid has become an emerging pesticide for the control of agricultural pests. When insects ingest imidacloprid, the nicotinic acetylcholine receptors (nAChRs) in their nerve synapses become disturbed; this hinders the normal transmission of nerve messages, which negatively influences the insects’ behavior [1]. Although imidacloprid is an extremely potent neurotoxic insecticide, it can also act as an nAChR activator [2]. Pharmacologic studies have shown that the blocking of acetylcholine receptors (AChRs) impairs memory formation, whereas acetylcholine promotes memory and learning functions. When AChRs in the hippocampus are damaged, severe deficits occur in spatial recognition and working memory [3]. However, in agriculture, pesticides such as imidacloprid are typically applied by spraying an entire area. As such, organisms other than insects may ingest them. In recent years, many studies have focused on the damage caused by neonicotinoid pesticides to nonpests such as honeybees. For instance, in honeybees, the ingestion of nectar containing imidacloprid and thiamethoxam may result in a major drop in honey production [4]. Moreover, excessive use of imidacloprid may reduce the number of wild honeybees because long-term exposure to imidacloprid has been noted to hamper the memory and learning functions of honeybees [5]. Furthermore, imidacloprid exposure may cause olfactory and visual dysfunction in honeybees in their larval stages [6]. As such, the use of neonicotinoid neurotoxic pesticides, such as imidacloprid, severely threatens the survival of pollinating insects such as honeybees, butterflies, and flower flies. For example, exposure to a small amount of imidacloprid can affect the flight path recognition ability of honeybees such that both the efficiency of their nectar collection as well as the number of bees returning to the hive decrease [7].

Many recent studies have reported the effects of imidacloprid exposure in vertebrates such as amphibians, fish, and mammals. Stacey et al. [8] observed that in the wood frog (*Lithobates sylvaticus*), exposure to neonicotinoid imidacloprid at 1, 10, and 100 μg/L during tadpole development may affect the ability of young frogs to sense or respond to predators. Vignet et al. [9] studied the effects of neonicotinoid pesticides on medaka and zebrafish development and behavior and observed that imidacloprid exposed for 5–14 days from 0.2 to 2000 μg/L had a sublethal effect on both types of fish. Burke et al. [10] found that in mice, exposure to imidacloprid (0.5 mg/kg/day) during pregnancy tended to result in lower fertility and body weight during adulthood, whereas exposure during developmental years negatively affected behavior and brain function during adulthood. Sriapha et al. [11] performed a case study on imidacloprid poisoning and found that imidacloprid with estimated doses of ingestion ranging from 2 to 35 g may increase the hepatic injury risk in humans. These results imply that imidacloprid-related injury during the embryo and infant stages may affect performance through adulthood and that even adult individuals can develop varying degrees of injury, including nonimmediate injury, after ingesting imidacloprid. Nerve repair is generally more difficult than muscle repair. For instance, damage to the cranial nerves during development can affect cognitive development to varying degrees. Tomizawa [12] reported that long-term exposure to imidacloprid (1 mg/kg/BW/day) may cause nAChR overexpression in the brain tissue of mammals, resulting in brain damage. In addition, Duzguner and Erdogan [13,14] reported that long-term exposure to 10 μM imidacloprid may induce inflammation and increase oxidative stress in the central nervous system of rats, eventually leading to neuronal apoptosis.

Insecticides can pose a serious threat to the survival of many animals in the wild. Neonicotinoid insecticides such as imidacloprid are widely used in agricultural production because they are easy to use, cheap, and effective. However, the excessive or incorrect use of pesticides may eventually result in water pollution. Therefore, in recent years, studies have increasingly used zebrafish as a model animal in experiments for detecting agricultural pesticides in the environment. In studies on the effects of pollution on water ecology, fish behavior patterns and the underlying neural transmission mechanisms have been employed to monitor water pollution and its potential impact on organisms. Zebrafish is one of the fish species recommended for aquatic acute toxicity testing by the OECD (Organization of Economic Cooperation and Development) not only because its developmental changes are easy to observe but also because it grows rapidly, is easy and inexpensive to raise, and has strong fecundity. In addition, the mechanism underlying its embryonic development is similar to that underlying mammalian embryonic development; it has various organ systems that are similar to those in humans, and its complete genetic database has been established [15]. As such, the zebrafish has become a major indicator organism in aquatic ecotoxicology, which can sensitively detect the toxicity of trace environmental pollutants. When the zebrafish is affected by environmental pollutants, its sensory system receives vital information, which passes through the central nervous system [16]. Systematic regulation of various physiological systems means that it exhibits behaviors such as swarming, aggression, fear, and vigilance [17]. Therefore, the behavior of zebrafish can be used as a crucial indicator in behavioral research. Several studies on imidacloprid have focused on embryonic development [18], but few studies have assessed adult individuals of vertebrates such as fish and mammals. In the present study, we determined the possible effects of imidacloprid exposure on the social behavior of adult zebrafish.

## 2. Materials and Methods

### 2.1. Animal Preparation

Adult wild-type zebrafish (*Danio rerio*) were obtained from Academia Sinica (Taipei, Taiwan). They were reared in circulating tap water at room temperature and were fed twice a day. Zebrafish, fish food, and other supplies were purchased from local pet shops (Taipei, Taiwan) for these studies. Zebrafish were kept in 10-gallon holding tanks and were fed live brine shrimp, microworms, and Tetramin staple food ad libitum. Experiments were conducted on 16-week-old adult zebrafish that were a useful animal model to study the effects of acute and chronic imidacloprid exposure on social behavior because it is a gregarious fish that can express behaviors such as swarming, aggression, fear, and vigilance. The behavioral performance of zebrafish was studied in the current experiments, which complied with guidelines of the IACUC and were approved by the IACUC of our university (protocol no.: No. 103018).

### 2.2. Imidacloprid Preparation and Treatment

Imidacloprid was purchased from Sigma-Aldrich (St. Louis, MO, USA; PESTANAL, analytical standard; concentration ≤ 100%; empirical formula (Hill Notation): C_9_H_10_ClN_5_O_2_; molecular weight: 255.66; CAS number: 138261-41-3). Imidacloprid is a neonicotinoid insecticide in the chloronicotinyl nitroguanidine chemical family. The International Union of Pure and Applied Chemistry (IUPAC) name is 1-(6-chloro-3-pyridylmethyl)-N-nitroimidazolidin-2-ylideneamine and the Chemical Abstracts Service (CAS) registry number is 138261-41-3. The molecular structure of imidacloprid is listed in Table 1. Imidacloprid powder was dissolved in 0.5% dimethyl sulfoxide (Sigma-Aldrich) as a cosolvent that was diluted with tap water to concentrations of 0.1, 0.5, and 1.0 ppm. During the observation experiment, which lasted 5 consecutive days, the solution was refreshed daily. The excrement in the fish tank was cleaned daily, and imidacloprid consumed during the experiment was supplemented, as required. Based on the fact that imidacloprid has an impact on the movement trajectory of zebrafish and does not cause fish death, we selected the initial concentrations of imidacloprid to be 0.1, 0.5, and 1.0 ppm; in order to avoid premature death of zebrafish in the 5-day experiment, a concentration of 0.1 ppm was chosen for the 5-day experiment. All the stock solutions were used at a room temperature of 25 ℃.

### 2.3. Locomotor Behavior Assay of the Zebrafish

The zebrafish were exposed to imidacloprid at different concentrations (0.1, 0.5, and 1.0 ppm) at different time points (0, 24, 48, 72, 96, and 120 h) for locomotion testing. Locomotor behavior of zebrafish was examined and tracked daily using a high-speed camera (Mikrotron-GmbH, Unterschleißheim, Germany) and were then analyzed using EthoVision-X (Noldus, Wageningen, The Netherlands). The behavioral performance of zebrafish in the current experiments was recorded and tracked daily for 20 min. Locomotor behavior assays of the zebrafish such as the swimming velocity and distance were examined using a high-speed camera.

### 2.4. Social Interaction Assay of the Zebrafish

We placed a female and a male zebrafish in two adjacent fish tanks. We recorded the movement trajectories of the female or male zebrafish in the separate fish tanks when a baffle plate was placed between the fish tanks to prevent the zebrafish from seeing each other. When the baffle plate was removed and the zebrafish saw each other in adjacent tanks, the movement trajectories of the female and male zebrafish were recorded again. In addition to recording the movement distance and speed and maximum acceleration and deceleration of the female and male zebrafish, we analyzed the relative distance between the female and male zebrafish, which was named the heterosexual attraction distance. Changes in this distance were considered to denote effects on the heterosexual attraction of the fish.

We placed one male zebrafish each into two adjacent fish tanks. We recorded the movement trajectories of the zebrafish in the separate fish tanks when a baffle plate was placed between the fish tanks. When the baffle plate was removed, the movement trajectories of the zebrafish were recorded again. In addition to recording the movement distance and speed and maximum acceleration and deceleration of both the zebrafish, we analyzed the relative distance between the two male zebrafish, which was named the vigilant confrontation distance. Changes in this distance were considered to denote effects on the defensive alert behavior of the fish.

### 2.5. Hematoxylin and Eosin Staining and Immunohistochemical Staining of Zebrafish Brain Tissue

Male zebrafish were anesthetized and perfused with PBS containing 4% formaldehyde (EM grade glutaraldehyde solution, Sigma-Aldrich). Brain tissue samples from male zebrafish were fixed with 4% formaldehyde (Sigma-Aldrich) and embedded in paraffin. Brain tissue specimens were cut into 5 μm thick sections using a tissue microtome, and then, sections were mounted on glass slides. Some brain tissue sections were stained with hematoxylin and eosin (H&E) (Sigma-Aldrich) to assess tissue integrity. Other myocardial tissue sections were subjected to immunohistochemical (IHC) staining with SOD2 (Cat. numbers ab110300; Abcam, Cambridge, UK), tumor necrosis factor (TNF)-α (Cat. numbers #3707; Cell Signaling Technology, Danvers, MA, USA), and caspase-3 (Cat. numbers #9662; Cell Signaling Technology) for 1 h at a room temperature of 25 ℃. By incubating with biotinylated secondary antibody (NovolinkTM Polymer Detection System l, Leica Biosystems Newcastle Ltd., Newcastle, UK) for 30 min and avidin–biotin–horseradish peroxidase (HRP) complex (Novolink™ Polymer Detection System l, Leica Biosystems Newcastle Ltd.) for an additional 30 min. IHC was visualized using DAB Chromogen (NovolinkTM Polymer Detection System 1, Leica Biosystems Newcastle Ltd.), and slides were counterstained with hematoxylin (NovolinkTM Polymer Detection System 1, Leica Biosystems Newcastle Ltd.).

### 2.6. Statistical Analysis

In this study, SigmaPlot 12.5 (Systat Software Inc., San Jose, CA, USA) was used for data analysis and chart production. All data are shown as mean ± standard error of the mean (SEM). Differences among different groups of zebrafish were assessed using one-way or two-way analysis of variance (ANOVA). Student–Newman–Keuls multiple comparisons post hoc test was performed if a significant F-value was obtained. Significance was defined as *p* < 0.05.

## 3. Results

### 3.1. Effects of Imidacloprid Exposure on Optic Tectum of Adult Zebrafish

**Figure 1:**
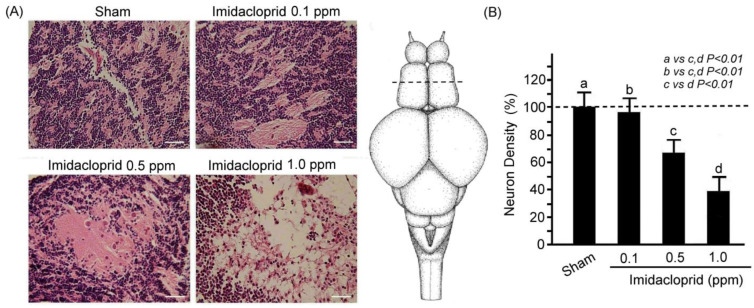
(**A**) H&E staining showing neuronal injury in telencephalon sections of an adult male zebrafish after 5 days of sham treatment and imidacloprid treatment at various concentrations. Scale bar = 25 µm. The dotted line shows transverse sections across a zebrafish adult brain in the telencephalon. (**B**) Neuron density in telencephalon sections of adult male zebrafish without (a: sham) and with imidacloprid (b: 0.1, c: 0.5, and d: 1.0 ppm) treatment after 5 days of exposure. Data are presented as mean ± SEM and were obtained from 10 independent experiments. One-way ANOVA followed by the Student–Newman–Keuls multiple comparison post-test. The differences in the median values among the treatment groups were greater than would be expected by chance; there is a statistically significant difference (*p* (Factor A) = 0.01).

Through H&E staining, we assessed neuronal injury in the optic tectum sections of the adult male zebrafish after 5 days of sham and imidacloprid exposure at various concentrations (Figure 1). We observed that the neuron number in the optic tectum sections was significantly higher in the adult zebrafish exposed to the sham treatment than in those exposed to imidacloprid (Figure 1A). Moreover, neuron density in these sections was significantly lower in adult zebrafish exposed to imidacloprid than in those that received the sham treatment (Figure 1B). Furthermore, as the imidacloprid concentration was increased, the neuron density decreased gradually and significantly (Figure 1B).

**Figure 2:**
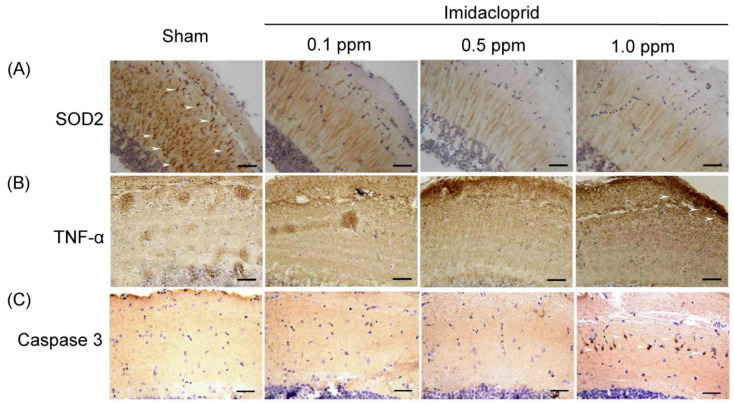
IHC staining showing neuronal (**A**) oxidative stress, (**B**) inflammation, and (**C**) apoptosis in telencephalon sections of adult male zebrafish without (sham) and with imidacloprid (0.1, 0.5, and 1.0 ppm) treatment after 5 days of exposure. The arrows denote positive expression of proteins related to neuronal antioxidative stress (SOD2), inflammation (TNF-α), and apoptosis (caspase-3). Scale bar = 100 µm.

Anti-oxidative stress SOD2 expressions were examined in the brain tissue of an adult male zebrafish using IHC staining and are shown in Figure 2A. SOD2 is a crucial antioxidant enzyme associated with oxidative stress. We observed that SOD2 protein expression in the brain tissue was weaker in the adult male zebrafish subjected to imidacloprid treatment than in those subjected to the sham treatment. Moreover, as the imidacloprid was concentration increased, SOD2 expression in the brain tissue of the adult male zebrafish exhibited a gradual decrease (Figure 2A).

TNF-α expression, an inflammation marker, in the brain tissue of an adult male zebrafish was examined and is shown in Figure 2B. We observed that TNF-α expression in the brain tissue of the adult male zebrafish subjected to imidacloprid treatment was higher than in those subject to the sham treatment. As the imidacloprid concentration was increased, TNF-α expression gradually increased in the brain tissue of the adult male zebrafish (Figure 2B).

Finally, caspase-3 expression, an apoptosis marker, in the brain tissue of an adult male zebrafish was examined, as shown in Figure 2C. We observed that caspase-3 expression in the brain tissue of the adult male zebrafish subjected to imidacloprid treatment was higher than in those subjected to the sham treatment. As the imidacloprid concentration increased, caspase-3 expression gradually increased in the brain tissue of the adult male zebrafish (Figure 2C). Taken together, these results confirmed that imidacloprid may cause neurotoxicity through oxidative stress, inflammation, and apoptosis in the brain tissue of the adult male zebrafish.

### 3.2. Effects of Acute and Chronic Imidacloprid Exposure on Locomotor Behavior of Adult Zebrafish

**Figure 3:**
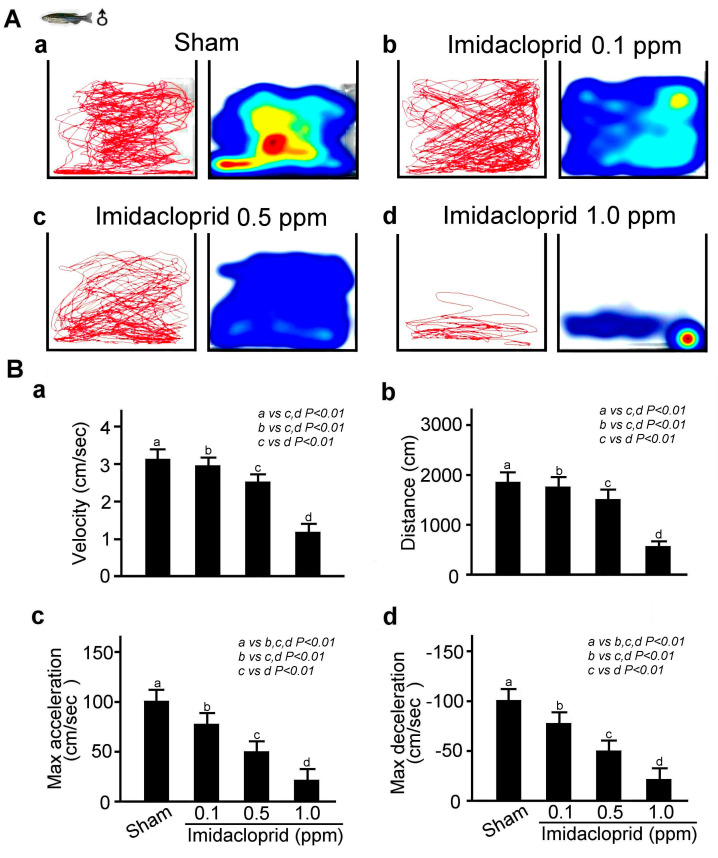
Locomotor behavior disability in adult male zebrafish exposed to imidacloprid for 24 h at various concentrations in a novel tank diving test. (**A**) Tracking and heat maps of the behavior trajectory of a male zebrafish without imidacloprid treatment (sham) and with imidacloprid treatment at various concentrations (0, 0.1, 0.5, and 1.0 ppm) after 24 h of exposure. (**B**) Bar charts showing swimming ability in quantified movement speed (**a**), movement distance (**b**), maximum acceleration (**c**), and maximum deceleration (**d**) in adult male zebrafish without imidacloprid treatment (sham) and with imidacloprid treatment at various concentrations (a: 0, b: 0.1, c: 0.5, and d: 1.0 ppm) after 12 h of exposure. Data are presented as mean ± SEM and were obtained from 10 independent experiments. The differences in the median values among the treatment groups were greater than would be expected by chance; there is a statistically significant difference (*p* (Factor A) = 0.01).

Using a Noldus Ethovision-XT animal behavior trajectory tracking analysis system, we analyzed the locomotor behavior of a single adult male zebrafish after they had spent 12 h in clean water and water environments containing different concentrations of imidacloprid. The movement trajectory map and movement trajectory heat map of the zebrafish were recorded for 10 min. According to the results shown in Figure 3A, compared with the zebrafish not exposed to imidacloprid, those exposed to different concentrations of imidacloprid moved more slowly and a shorter distance and had lower maximum acceleration and deceleration after 12 h; the nonimidacloprid group exhibited the best locomotor behavior, whereas the group with the highest imidacloprid exposure exhibited the worst locomotor behavior. These results are corroborated by the bar charts shown in Figure 3B (*p* < 0.01 or 0.05).

**Figure 4:**
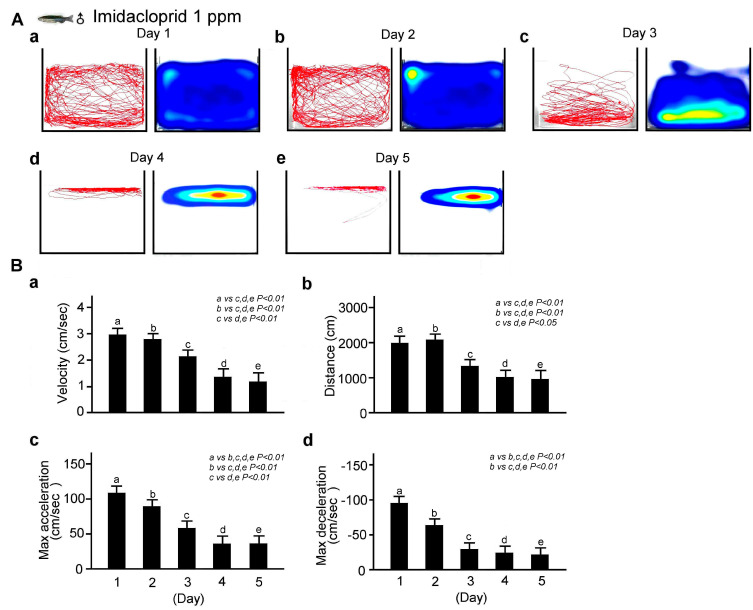
Locomotor behavior disability in adult male zebrafish exposed to 0.1 ppm imidacloprid for various exposure times in a novel tank diving test. (**A**) Tracking and heat maps of the behavior trajectory of male zebrafish without imidacloprid treatment (sham) and with 0.1 ppm imidacloprid treatment after various exposure times (1, 2, 3, 4, and 5 days). (**B**) Bar charts showing swimming ability in quantified movement speed (**a**), movement distance (**b**), maximum acceleration (**c**), and maximum deceleration (**d**)—in adult male zebrafish without imidacloprid treatment (sham) and with 0.1 ppm imidacloprid treatment after various exposure times (a: 1, b: 2, c: 3, d: 4, and e: 5 days). Data are presented as mean ± SEM and were obtained from 10 independent experiments. The differences in the median values among the treatment groups were greater than would be expected by chance; there is a statistically significant difference (*p* (Factor A) = 0.01).

We next explored the changes in locomotor behavior of adult male zebrafish immersed in a fixed concentration (0.1 ppm) of imidacloprid for different exposure durations (1, 2, 3, 4, or 5 days). As shown in Figure 4A, the locomotor behavior of the male zebrafish after exposure to 0.1 ppm imidacloprid for 5 consecutive days was recorded using our tracking analysis system. We observed that the longer the exposure time, the more significant the decrease in the average movement distance and speed as well as maximum acceleration and deceleration. These results are corroborated by the bar charts shown in Figure 4B (*p* < 0.01 or 0.05).

### 3.3. Effects of Imidacloprid Exposure on Social Interaction of Adult Zebrafish

**Figure 5:**
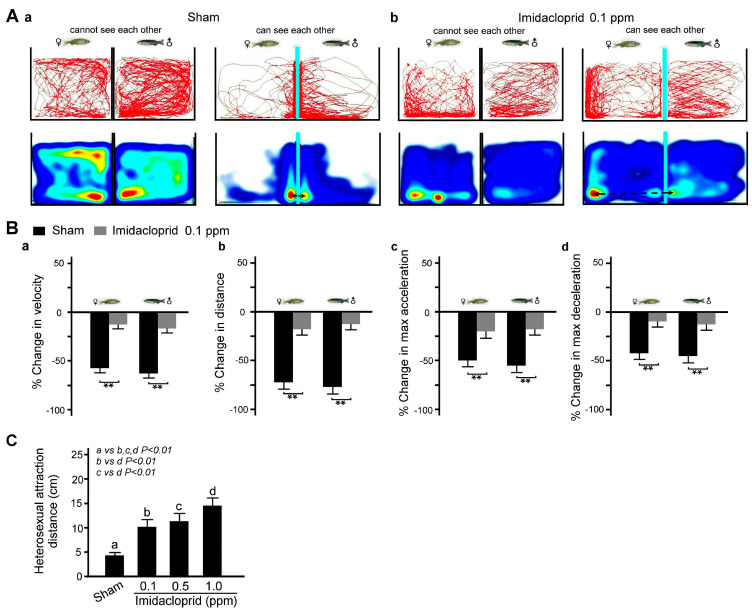
Heterosexual attraction disability in adult male and female zebrafish after 5 days of imidacloprid exposure in a novel tank diving test. (**A**) Tracking and heat maps of the behavior trajectory of a male and a female zebrafish without imidacloprid treatment (sham) and with 0.1 ppm imidacloprid treatment for 5 days. The dotted lines denote the heterosexual attraction distance. (**B**) Bar charts showing swimming ability in quantified movement speed (**a**), movement distance (**b**), maximum acceleration (**c**), and maximum deceleration (**d**) in adult male and female zebrafish without imidacloprid treatment (sham) and with 0.1 ppm imidacloprid treatment for 5 days. (**C**) Bar chart showing quantified heterosexual attraction distance without imidacloprid treatment (sham) and with imidacloprid treatment (a: 0, b: 0.1, c: 0.5, and d: 1.0 ppm) for 5 days. Data are presented as mean ± SEM (n = 10 for each group, ** *p* < 0.01).

We further examined the effects of imidacloprid exposure on social interaction in adult zebrafish by analyzing the movement trajectories of zebrafish in two adjacent fish tanks. As shown in Figure 5A, we found that in a clean water environment, the movement distance and speed and maximum acceleration and deceleration were similar for the male and female zebrafish in separate tanks when the baffle plate was present; however, they then decreased when the baffle plates were removed, indicating heterosexual attraction. However, after exposure to 0.1 ppm imidacloprid, the heterosexual attraction between the female and male fish was significantly weaker (Figure 5B, *p* < 0.01 or 0.05).

**Figure 6:**
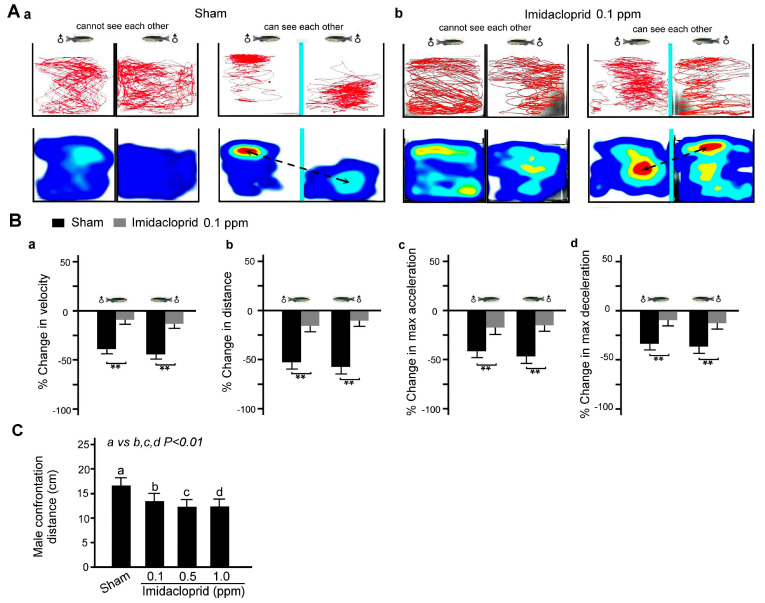
Defensive alert behavior disability in two adult male zebrafish after 5 days of imidacloprid exposure in a novel tank diving test. (**A**) Tracking and heat maps of the behavior trajectory of two male zebrafish without imidacloprid treatment (sham) and with 0.1 ppm imidacloprid treatment for 5 days. The dotted lines denote the vigilant confrontation distance. (**B**) Bar charts showing swimming ability in quantified movement speed (**a**), movement distance (**b**), maximum acceleration (**c**), and maximum deceleration (**d**) in two adult male zebrafish without imidacloprid treatment (sham) and with 0.1 ppm imidacloprid treatment for 5 days. (**C**) Bar chart showing quantified confrontation distance without imidacloprid treatment (sham) and with imidacloprid treatment (a: 0, b: 0.1, c: 0.5, and d: 1.0 ppm) for 5 days. Data are presented as mean ± SEM (n = 10 for each group; ** *p* < 0.01).

Two male zebrafish were placed one each in adjacent fish tanks, and the zebrafish could not see each other due to a baffle plate between the tanks. As shown in Figure 6A, we found that in a clean water environment, the movement distance and speed as well as maximum acceleration and deceleration of two male zebrafish in separate tanks were similar when the baffle plate was employed; additionally, they decreased when the baffle plate was removed, indicating defensive alert behavior because the behavior of male zebrafish in the two tanks is compared to a defensive alert. However, after exposure to imidacloprid, this behavior was significantly decreased (Figure 6B).

## 4. Discussion

To adapt to their environment and survival needs, fish require skills such as spatial learning and predator evasion so as to identify and avoid predators as well as find suitable breeding grounds. We found that regardless of their sex, the movement distance and speed as well as maximum movement acceleration and deceleration of adult zebrafish were significantly altered by imidacloprid exposure; the longer the imidacloprid exposure, the more severe the locomotor behavior disability. Moreover, imidacloprid significantly reduced the heterosexual attraction between an adult male and an adult female zebrafish and significantly affected defensive alert behavior between two adult male zebrafish. Because the zebrafish is a gregarious fish, it displays behaviors such as grouping, aggression, fear, and vigilance; different nuclei in the brain dominate these social behaviors. Our results demonstrated that imidacloprid exposure significantly negatively affected the performance of social behaviors such as mutual attraction and mutual alertness in zebrafish, indicating that exposure to a systemic pesticide such as imidacloprid may damage the neural nuclei in the brain related to emotion or courtship behavior.

The forebrain of the goldfish develops into the telencephalon, a brain area responsible for cognitive learning and emotional regulation. Moreover, in goldfish, damage to the outer telencephalon causes disturbance in spatial memory [19], whereas that to the inner telencephalon leads to disturbance in emotional memory [20]. The goldfish telencephalon is equivalent to the limbic system of mammals in that the function of the medial telencephalic pallium is equivalent to that of the amygdala in the limbic system—the nucleus that controls emotional memory. If the medial telencephalic pallium is damaged, goldfish lose memory of their past experiences of fear [20]. Furthermore, the function of the lateral telencephalic pallium in goldfish is similar to that of the hippocampus, which controls spatial cognition and emotional expression [21]. The zebrafish has gradually been developed as an animal model for exploring cognitive functions, and some studies have indicated that it has the ability to learn and remember fear experiences [22]. In addition, the administration of nicotine receptor agonists to zebrafish can improve cognitive learning [23,24].

The ADME (absorption, distribution, metabolism, and elimination) of imidacloprid exposure has been reported in many studies. Imidacloprid is rapidly and completely absorbed and distributed after oral administration in rats; after exposure, the peak plasma level of imidacloprid is reached within 2 h, whereas the peaks in liver, kidney, lung, and skin levels occur after 48 h in rats [25]. Imidacloprid is mainly metabolized in the liver and then excreted through urine. Imidacloprid can be oxidatively cleaved into 6-chloronicotinic acid, which is further metabolized through glutathione binding to form mercaptonicotinic acid and hippuric acid. In addition, imidacloprid can be metabolized through imidazolidine ring hydroxylation, thus generating 5-hydroxyl and alkene derivatives [26]. Approximately 90% of imidacloprid is eliminated within 24 h of exposure: 75–80% through urine and 10–15% through feces via biliary excretion [27]. The differences between species are believed to be due to differences in metabolic rates and associated cumulative oxidative damage. Therefore, different species demonstrate differing changes in the pharmacokinetics of ADME and other aspects of susceptibility to hazards associated with a toxicant such as imidacloprid.

Imidacloprid is a highly potent neurotoxic insecticide; it blocks AChRs in hippocampal neurons and impairs memory formation [3]. Long-term exposure to imidacloprid may damage neurons in the brain and impair the memory and learning functions of honeybees [5]. We previously reported that in bats, imidacloprid exposure can cause neuronal apoptosis in the CA1 area of the hippocampus and the medial entorhinal cortex related to memory learning in the brain, which in turn triggers spatial memory impairment [28]. In addition, we previously revealed that inflammation and mitochondrial-dysfunction-related apoptosis in the hippocampal CA1 and medial entorhinal cortex areas may induce orientation disorder and spatial memory dysfunction [29]. In the current study, the results of our histomorphology and IHC staining using brain tissue sections indicated that imidacloprid exposure causes neuronal oxidative stress, inflammation, apoptosis, and damage in the telencephalon of adult zebrafish. Extremely high imidacloprid concentrations have been reported to induce a considerable increase in reactive oxygen species (ROS) levels in the zebrafish liver and enhance the activities of SOD, CAT, and GST, which scavenge excess ROS. ROS attack cell membranes, leading to an increase in MDA, which causes DNA damage and increases GST scavenging activity [30].

## 5. Conclusions

**Figure 7:**
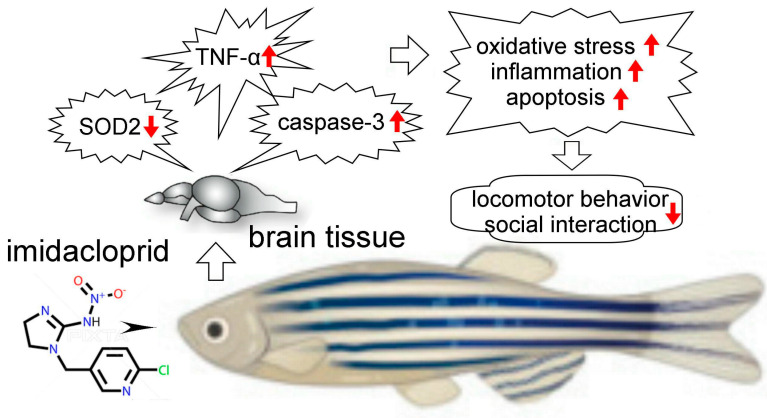
Possible pathological mechanism underlying brain damage induced by imidacloprid in zebrafish. Our behavioral and histological experiments showed that adult zebrafish exposure to the neonicotinoid imidacloprid may affect the performance of zebrafish social behavior because imidacloprid may damage neurons in the telencephalon of zebrafish.

The neonicotinoid imidacloprid is considered to be one of the most commonly detected neonicotinoid insecticides in surface waters. Imidacloprid concentrations in surface waters range from 0.001 to 320 ppb, while some estimates of accidental spills can even be as high as 1.8 to 7.3 ppm, which can adversely affect aquatic organisms [31,32]. Our behavioral and histological experiments showed that adult zebrafish exposure to the 0.1–1.0 ppm neonicotinoid imidacloprid can affect the performance of zebrafish social behavior, which we believe is partly due to imidacloprid damaging neurons in the telencephalon of zebrafish through oxidative stress, inflammation, and apoptosis. (Figure 7). From the results of this experiment, we believe that the behavioral responses of zebrafish can be used as a biomarker for assessing environmental toxicological risks.

## Figures and Tables

**Table 1:** Molecular structure, molecular formula, and molecular weight of imidacloprid.

Molecular structure	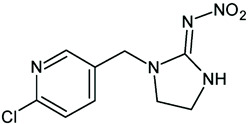
Molecular formula	C_9_H_10_ClN_5_O_2_
Molecular weight	255.662 g/mol

## Data Availability

Not applicable.
